# To oral health and beyond: integrating maternal-child oral health to prevent early childhood caries

**DOI:** 10.3389/fdmed.2026.1884801

**Published:** 2026-10-30

**Authors:** Giovanna Torqueto Castilho, Caroline Meronha de Lima, Marilia Narducci Pessoa, Elaine Pereira da Silva Tagliaferro, Ramiro Mendonça Murata, Vanessa Pardi

**Affiliations:** 1 Department of Morphology and Pediatric Dentistry, São Paulo State University (UNESP), School of Dentistry, Araraquara, São Paulo, Brazil; 2 Department of Community Dentistry, São Paulo State University (UNESP), School of Dentistry, Araraquara, São Paulo, Brazil; 3 Department of Foundational Sciences, School of Dental Medicine, East Carolina University, Greenville, NC, United States

**Keywords:** caries prevention, digital health, early childhood caries, maternal oral health, maternal-child health, oral microbiome, pediatric dentistry

## Abstract

Early childhood caries (ECC) remains one of the most prevalent chronic diseases affecting children worldwide. Increasing evidence suggests that maternal biological, behavioral, and developmental factors during pregnancy influence infant oral microbiome establishment and early caries susceptibility before clinical disease becomes evident. Prenatal oral healthcare, maternal oral health education, and caregiver-centered interventions have therefore emerged as important components of preventive strategies. This Perspective synthesizes current evidence on maternal–infant transmission dynamics and proposes the Three-Dimensional Maternal Axis as a conceptual framework for understanding early-life influences on ECC development. Building on prior models of maternal influence on childhood caries, this framework foregrounds biological, behavioral, and developmental influences as proximal and interconnected pathways operating across pregnancy and early childhood. A critical preventive window appears to extend from pregnancy through approximately three years of age, during which enamel development, microbial colonization, feeding transitions, and oral health behaviors become established. The article further discusses the translational implications of maternal-centered prevention approaches, including integration of oral health into prenatal care, continuity of care during early childhood, and the potential role of digital health technologies in expanding access to preventive support. Particular attention is given to implementation challenges, health equity, and opportunities for interdisciplinary and cross-sector prevention models. Together, these findings support broader and earlier approaches to ECC prevention that integrate maternal-child oral health across developmental stages and caregiving contexts.

## 1 Introduction

Mothers have long been recognized as an important source of cariogenic bacteria during early childhood, with *Streptococcus mutans* and other microorganisms commonly transferred through routine caregiving practices (1–3). Yet most preventive strategies continue to focus primarily on the child after tooth eruption (4, 5), creating a fundamental disconnect between what is known about early microbial acquisition and when prevention begins.

Early childhood caries (ECC) remains one of the most prevalent chronic diseases affecting children worldwide, with substantial implications for quality of life and healthcare systems (4, 5). Despite decades of evidence linking maternal oral microbiota to initial colonization of the infant oral cavity, preventive models have been slow to incorporate this knowledge. In a cohort of 448 mother–infant pairs, maternally derived bacteria accounted for a median of 9.7% of the infant oral microbiota at four months, significantly exceeding microbial sharing with unrelated mothers, and notably, formula-fed infants acquired more maternal oral bacteria than exclusively breastfed infants, an association that held independent of delivery mode or antibiotic exposure, suggesting that feeding practices may play a role in shaping microbial establishment (2). Systematic reviews using sequencing technologies confirm that maternal microbial communities contribute substantially to early oral microbial exposure, with the infant oral microbiome undergoing rapid ecological changes shaped by feeding, environment, host development, and caregiver interactions during the first months of life (1). Recent translational studies further suggest that maternal oral pathobionts transmitted to the infant gut may modulate intestinal immunity and increase susceptibility to inflammatory conditions later in life, even after these microorganisms are no longer detectable (6).

However, maternal influences on ECC extend well beyond microbial transfer. Dietary habits, oral health literacy, hygiene behaviors, and prenatal factors such as vitamin D status have all been associated with caries risk in children (7, 8). We argue that these biological, behavioral, and developmental pathways are not isolated mechanisms but interconnected dimensions of a single maternal influence that operates across pregnancy and early childhood. In this paper, we propose the “Three-Dimensional Maternal Axis” as a conceptual framework integrating these dimensions into a unified model for ECC prevention, not to replace established child-focused measures, but to address the critical gap between what the evidence reveals about early-life risk and what current preventive strategies target. We also examine potential applications of this framework in digital health and population-based strategies.

## 2 The three-dimensional maternal axis

Understanding maternal influences on early childhood caries requires considering interconnected biological, behavioral, and developmental pathways rather than isolated mechanisms. Building on prior models that emphasize social-environmental mediators of maternal influence on caries risk (9), we propose the “Three-Dimensional Maternal Axis” framework, which foregrounds biological, behavioral, and developmental influences as proximal pathways, situated within-rather than equated with- broader social and environmental contexts that collectively shape early oral health trajectories and ECC susceptibility (Figure 1).

**Figure 1:**
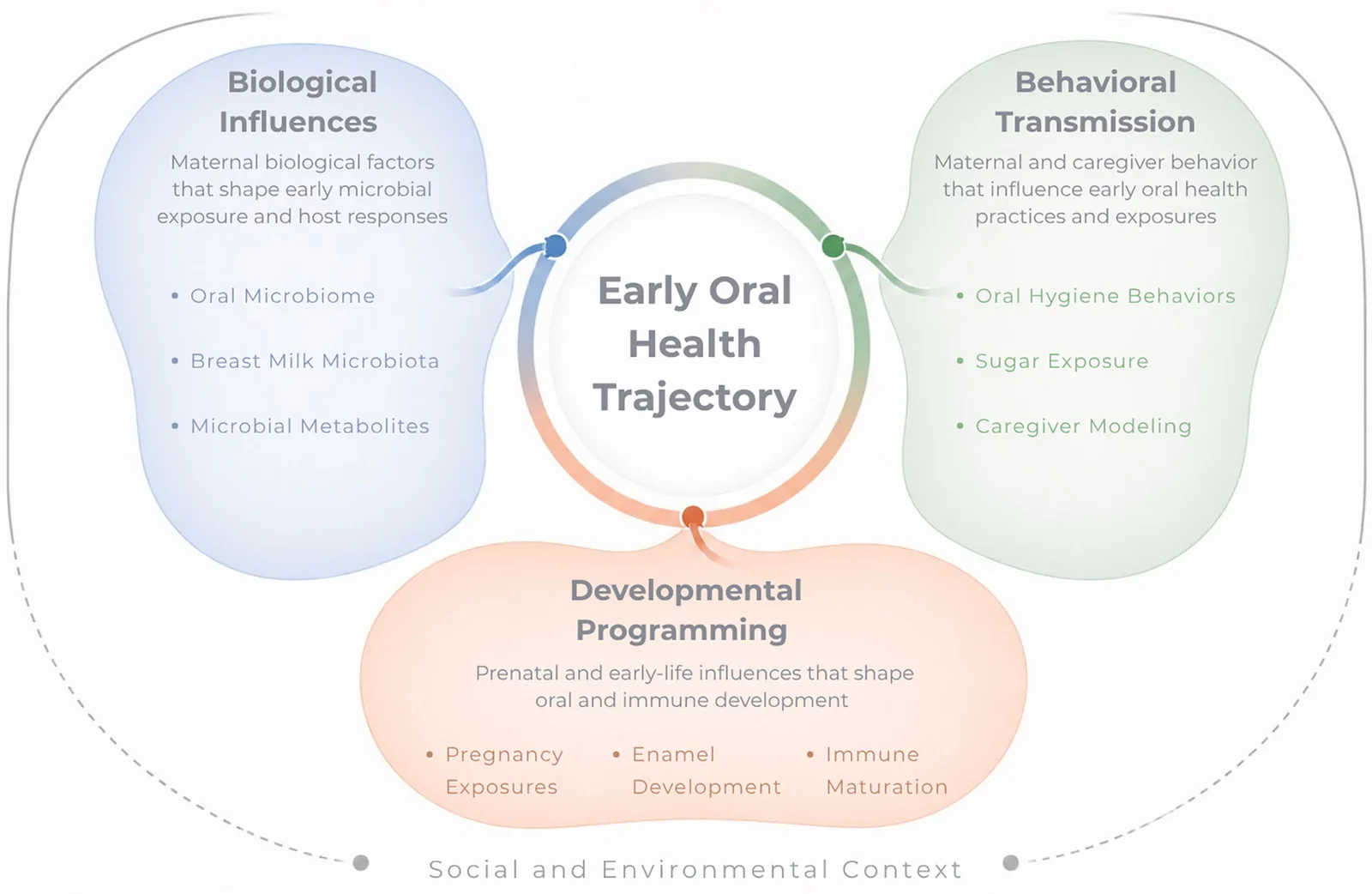
The three-dimensional maternal axis framework integrating biological influences, behavioral transmission, and developmental programming as proximal pathways shaping early oral health trajectories and ECC susceptibility within broader social and environmental contexts (Created with Canva - canva.com).

### 2.1 Biological influences

Maternal biological systems, including the gut, vaginal tract, breast milk, and oral cavity, contribute microorganisms, metabolites, and environmental signals that influence the establishment of infant microbial ecosystems with potential downstream effects on immune maturation and metabolic signaling (10). Rather than passive transfer, these interactions involve dynamic host–microbe processes. Microbial metabolites, including short-chain fatty acids and indole derivatives, may influence gene regulation through histone modification, DNA methylation, and noncoding RNA activity (10). Beyond microbial communities, emerging proteomic evidence suggests the biological axis may extend to shared molecular signatures as well. A longitudinal profiling study of a small cohort of mother-infant dyads identified overlapping salivary proteins, including amylase isoforms and oligosaccharide metabolism, across pregnant women, postpartum mothers, and their one-month-old infants, suggesting that the biological axis may operate through multiple molecular layers beyond microbiome composition alone (11).

Population-based studies reinforce this picture. In cohorts of pregnant women, partners, and infants, maternal biological factors contributed substantially to infant microbiota composition, with transmission routes varying by mode of delivery. Vaginally born infants exhibited greater representation of microbial taxa associated with maternal fecal communities, whereas cesarean-born infants demonstrated reduced fecal microbiota transfer but increased colonization by breast milk–associated taxa, a pattern that may represent a compensatory mechanism offsetting the reduced exposure to maternal fecal microbial communities (3, 12).

Data from mother–partner–infant triads further indicate that vaginal delivery is associated with taxa also identified in maternal oral samples; however, these patterns suggest ecological overlap rather than evidence of direct maternal oral influence on infant microbial assembly (13). Longitudinal evidence from a small cohort of mother–infant dyads further demonstrate that the maternal salivary microbiome remains compositionally stable from the third trimester through six months postpartum, while infant oral communities undergo dynamic ecological succession, initially dominated by *Streptococcus* and progressively converging toward maternal profiles, with cariogenic genera persisting throughout this critical window (14).

Preterm birth has been linked to lower oral microbiome diversity and increased abundance of potentially pathogenic taxa (15). Similarly, maternal inflammatory state and lower socioeconomic position have been independently associated with increased alpha diversity of the neonatal buccal microbiota. Although the original study attributed this to transient maternal microbial transfer we propose this pattern may alternatively reflect early dysbiosis community assembly rather than a beneficial increase in microbial richness, consistent with reports linking higher oral diversity to periodontitis and opportunistic pathogen colonization (16).

### 2.2 Behavioral

Maternal oral health literacy, dietary practices, hygiene behaviors, and healthcare utilization have all been associated with caries outcomes in young children (7). These pathways operate in interconnected ways: maternal oral health practices may influence bacterial transmission while simultaneously shaping behaviors related to sugar consumption, hygiene, and healthcare engagement.

Feeding practices illustrate this interaction. Breastfeeding during the first year has been associated with lower caries risk, although findings become more heterogeneous beyond 12 months (7), likely reflecting multiple overlapping mechanisms including feeding behaviors, delayed bottle introduction, and reduced free sugar exposure, working in concert with the antimicrobial and prebiotic properties of breast milk (7, 17). Beyond its nutritional role, human milk contributes microbial communities, human milk oligosaccharides, and antimicrobial proteins that actively participate in shaping the infant oral microbiome (17).

Maternal dietary habits represent a distinct pathway: sugar consumption patterns are established within the household environment and transmitted intergenerationally, and maternal added sugar intake was directly associated with greater sugar consumption by children, explaining chronic oral disease burden in early childhood (18). The timing of sugar introduction into the infant diet, a maternally controlled decision, carries measurable consequences. In the 2015 Pelotas Birth Cohort (*n* = 3,654), caries experience was 48% greater in children with sugar introduction before 12 months compared to those where sugar was introduced after 24 months (19). Importantly, early sugar exposure also reshapes the infant oral microbiome toward aciduric and acidogenic taxa, illustrating how the behavioral axis directly modulates the biological axis (1).

### 2.3 Developmental programming

Vitamin D exemplifies how the prenatal environment shapes the offspring caries risk trajectory. Population-based studies link higher maternal vitamin D levels—most consistently during the second and third trimesters, the window of active primary-tooth mineralization—with lower caries prevalence in offspring (8). Notably, cord-blood vitamin D deficiency at birth has been associated with a two-fold increase in dmft scores compared to non-deficient levels; however maternal prenatal vitamin D status (sufficient versus insufficient) alone was not significantly associated with caries outcomes in the same cohort (21), suggesting the neonatal/cord-blood measure may be a more sensitive predictor than maternal status *per se*. Biologically, vitamin D supports the calcium-phosphate homeostasis required for enamel mineralization and acts directly on ameloblasts and odontoblasts through vitamin D receptors that regulate enamel matrix proteins, while also driving antimicrobial peptide production in oral tissues to reinforce innate defense (8, 22, 23). However, evidence is not uniform; a large European cohort (Generation R) reported only a weak prenatal signal with significant associations limited to early-childhood rather than prenatal vitamin D levels, and Mendelian-randomization analyses did not support causation, cautioning against supplementation for caries prevention alone (24).

This inconsistency suggests vitamin D is best understood not as an isolated determinant, but as one node within a broader developmental pathway converging on the enamel substrate itself. Developmental defects of enamel, particularly enamel hypoplasia, represent a second, more direct prenatal programming pathway. Meta-analytic evidence indicates that children with enamel hypoplasia have approximately five-fold higher odds of developing caries in the primary dentition (RR 5.45; 95% CI 1.84–16.14), while any enamel defect roughly doubles caries risk (RR 1.94; 95% CI 1.52–2.49), a dose-response association reproduced across independent syntheses (25, 26). These defects have themselves been linked to prenatal factors including maternal vitamin D and retinol deficiency, prematurity, and low birth weight (27).

Beyond micronutrients, delivery mode, maternal antibiotic exposure, obesity, and diabetes further shape the intrauterine environment and the maternal/neonatal oral microbiome (10, 15, 20). Gestational diabetes, for instance, has been shown in animal models to act directly on tooth development: maternal hyperglycemia activates TLR4/NF-*κ*B signaling in offspring dental papilla cells, suppressing SMAD1/5/9 phosphorylation and inhibiting odontoblastic differentiation and dentin formation (28, 29). Taken together, these findings position vitamin D not as a discrete causal factor but as a marker of a broader, integrated developmental milieu, one whose final common pathway is a more caries-susceptible enamel substrate.

## 3 The critical window

The preventive window for ECC extends from late pregnancy through the first years of life, a period during which three three axes of the Maternal Axis framework converge to shape early oral health trajectories (Figure 2). This window maps closely onto the first 1,000 days framework, from conception through age two, already recognized in nutrition and child development policy as a period of heightened biological plasticity with lasting health consequences (30, 31). Extending this concept to oral health creates a policy-relevant rationale for integrating caries prevention into existing maternal-child health infrastructure.

**Figure 2:**
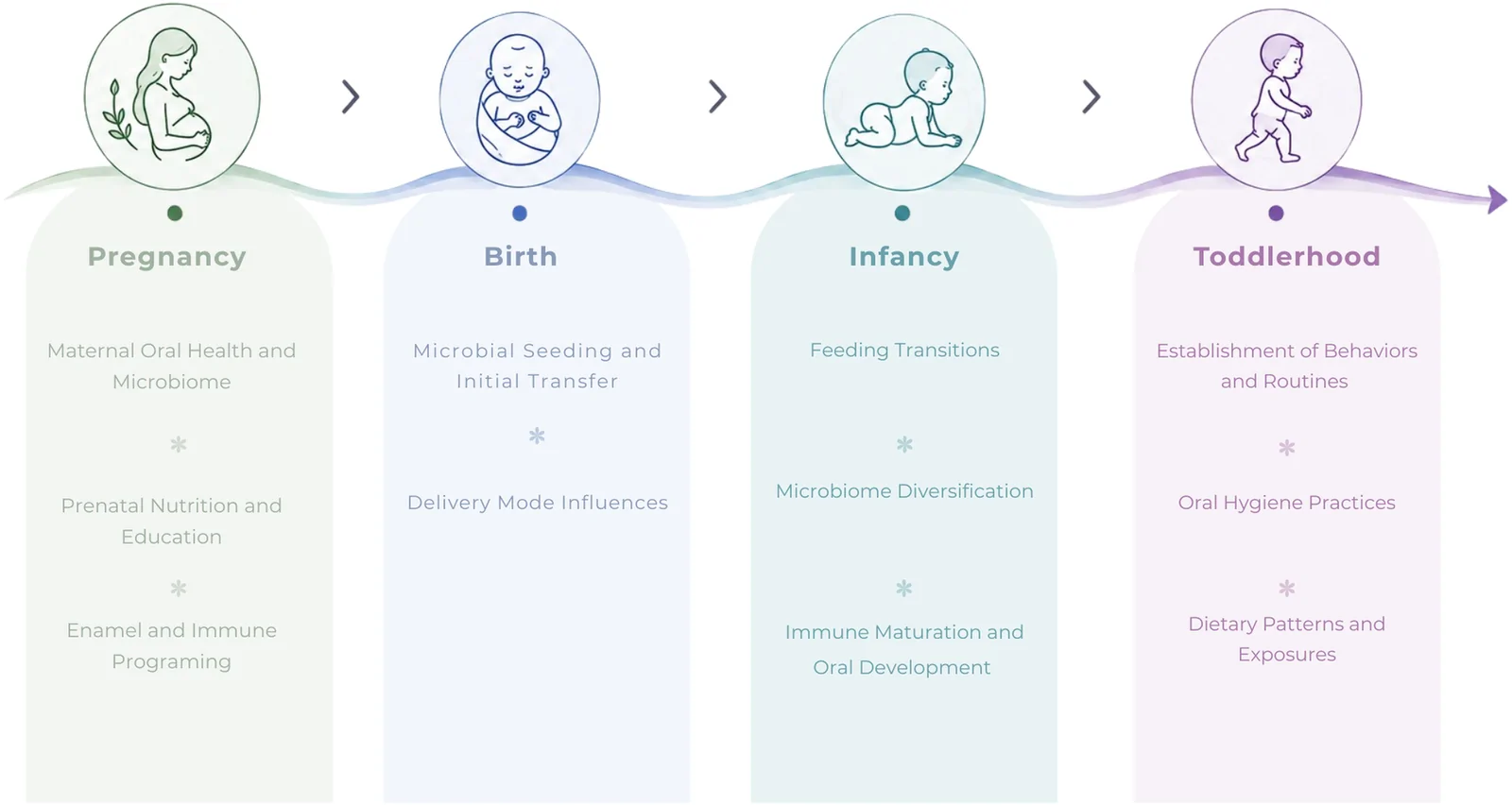
The Critical Window for ECC Prevention across the first 1,000 days, from pregnancy through toddlerhood, highlighting key biological, behavioral, and developmental events that shape enamel formation, oral microbiome establishment, and early caries susceptibility (Created with Canva - canva.com).

Late pregnancy, particularly the third trimester, represents an important developmental phase associated with offspring oral health outcomes through the developmental programming axis. Pregnancy is also a time when women are typically engaged with healthcare systems and may be receptive to preventive guidance, creating an opportunity to address the behavioral axis. Dental care during pregnancy is considered safe, and intervention studies suggest that preventive strategies initiated during pregnancy and maintained during infancy may improve early oral health outcomes (7, 32).

This temporal advantage is supported by the Baby Teeth Talk RCT, in which Aboriginal Australian children whose mothers received a multi-component intervention beginning during pregnancy had lower caries experience in the primary dentition at age 9 years compared to children who received the same intervention starting at age 2 (33, 34). Notably, the delayed intervention group still benefited relative to no intervention, but the earlier start conferred a persistent advantage, reinforcing the importance of the pregnancy-to-infancy window.

Between approximately six months and three years of age, developmental transitions such as tooth eruption, complementary feeding, and establishment of oral hygiene routines occur alongside rapid microbiome diversification and early community assembly (7). We contend that this convergence of biological, behavioral, and developmental transitions within a defined temporal window demands a restructuring of when and where preventive resources are deployed, from post-eruption intervention to a continuum beginning in pregnancy.

## 4 Evidence for maternal-centered prevention

The evidence for maternal-centered ECC prevention can be organized along the three axes of the proposed framework, revealing both the strengths and limitations of current approaches.

### 4.1 Biological axis

Prenatal oral health programs aimed at improving maternal oral status have been associated with lower caries risk in offspring (7, 35). Interventions including direct maternal oral healthcare have demonstrated more consistent benefits than educational approaches alone, consistent with the premise that reducing maternal bacterial burden modifies the biological transmission pathway, though antimicrobial treatments targeting maternal dentition alone have not demonstrated caries reduction in offspring (7), suggesting the mechanism extends beyond simple bacterial suppression. Advances in microbiome research may further refine this axis: oral microbiota composition has been associated with future ECC development, and early microbial dysbiosis has been detected prior to clinical disease onset, suggesting opportunities for microbiome-informed risk stratification (36–38).

### 4.2 Behavioral axis

The most comprehensive evidence syntheses indicate that interventions involving pregnant women and new mothers, particularly those combining diet and feeding practice guidance with direct clinical preventive care, are associated with modest but relatively consistent reductions in ECC (7). However, improvements in maternal knowledge do not consistently translate into measurable caries reduction, pointing to the insufficiency of education alone when structural barriers remain unaddressed (7, 35). Health literacy, cultural context, socioeconomic constraints, and the difficulty of sustaining behavioral change over time all shape how prevention messages are received and acted upon and none of these are resolved by knowledge-based approaches in isolation. Translating the behavioral axis into practice therefore depends less on what information is delivered than on how, how often, and in what combination with clinical care it reaches caregivers across the continuum prenatal-to-infancy.

This limitation is compounded by the structural determinants that shape how prevention messages are received and acted upon. Health literacy, cultural context, and socioeconomic background vary widely across populations (7, 39), meaning that a single counseling session, however well-designed, is unlikely to produce lasting change for most caregivers. Effective behavioral support requires repeated touchpoints distributed across the prenatal-to-infancy continuum, with content that evolves alongside the child's development: feeding practices and sugar introduction timing in early infancy, toothbrushing initiation around first tooth eruption, and fluoride use as the dentition establishes (5, 7, 39).

Digital tools like SMS reminders, anticipatory guidance applications, and web-based platforms, offer one avenue for extending this support between clinical visits. Evidence links mobile health interventions to reductions in plaque indices and white spot lesion incidence (40), though improvements have been more consistently observed among higher-resourced populations (41), raising equity concerns that parallel those seen across the broader implementation literature. Complex digital platforms remain aspirational in scope and are not yet supported by sufficient evidence to function as standalone strategies. Their role is best understood as complementary, reinforcing messages initiated in clinical encounters rather than replacing the contact itself (40, 41).

### 4.3 Developmental programming axis

A 2025 prospective cohort study reported inverse associations between maternal vitamin D levels during pregnancy and offspring ECC odds, with protective associations most pronounced in the second and third trimesters (8). However, a 2021 Dutch cohort study found that only early childhood vitamin D status was associated with caries risk after adjustment, and genetic analyses did not support a causal relationship (24). The observational evidence is suggestive but conflicting, and available trial data do not demonstrate a measurable benefit, suggesting evidence remains insufficient to support routine vitamin D supplementation specifically for ECC prevention. This axis remains the least developed in terms of translatable interventions, representing an important direction for future research.

Established pediatric preventive measures, including fluoride varnish application and fluoride supplementation, remain essential components of comprehensive ECC prevention (5). The Maternal Axis framework does not propose replacing these measures but rather extending the preventive timeline upstream to address risk factors that operate before and alongside conventional child-focused approaches.

## 5 Implementation: technology, equity and policy

### 5.1 Operationalizing the maternal axis in routine care

The Three-Dimensional Maternal Axis can be operationalized through coordinated preventive contacts across pregnancy and early childhood. During pregnancy, prenatal and dental providers can assess maternal oral health and facilitate dental referral when oral disease, symptoms, or unmet dental-care needs are identified (32, 42). Current guidance supports the safety of dental treatment during pregnancy and the incorporation of oral health assessment into prenatal care (32, 42, 43). During infancy, pediatric and primary-care providers can reinforce feeding guidance, oral hygiene, fluoride use, and establishment of a dental home, while incorporating oral health risk assessment into routine preventive visits (4, 5, 7). Following tooth eruption, dental referral should be prompted when caries risk is elevated, visible enamel abnormalities or suspected carious lesions are identified, or a dental home has not been established according to recommended preventive care schedules (5, 7).

Referral should function as a coordinated process across the prenatal-to-infancy continuum rather than as a single transfer of care. Prenatal, pediatric, primary-care, and dental providers can communicate relevant risk information and reinforce consistent prevention messages (39, 42). In this model, the Maternal Axis does not require a separate clinical pathway; rather, it provides a structure for integrating maternal and early-childhood risk assessment into existing preventive encounters. Specific screening and referral thresholds should be adapted to established clinical guidelines, local healthcare infrastructure, and population needs.

### 5.2 Equity, technology, and policy considerations

ECC remains disproportionately concentrated among children from low-income families, racial and ethnic minority populations, and Indigenous communities (44, 45). Equity should therefore be considered a foundational component of implementation rather than an additional consideration. Maternal-centered approaches may leverage existing healthcare contact points, particularly during pregnancy, to expand access to oral health counseling, screening, and referral while addressing misconceptions regarding the safety of dental treatment during pregnancy (32, 42). In Indigenous communities, community-based participatory approaches may further improve cultural relevance, acceptability, and sustainability of prevention strategies (45).

Digital health technologies may complement these efforts by extending preventive education, follow-up, and access to screening beyond conventional clinical settings. Teledentistry, digital messaging, video-based interventions, and other remote approaches may support preventive behaviors and facilitate contact with oral health services. However, differences in connectivity, device access, and digital literacy may limit their reach and potentially reinforce existing disparities. Digital approaches should therefore be considered complementary to, rather than replacements for, in-person care, with implementation adapted to local infrastructure and population needs.

Health policy and reimbursement structures also influence the feasibility and sustainability of maternal-centered prevention. In the United States, only about one-third of Medicaid-enrolled pregnant women report receiving a dental cleaning during pregnancy, and coverage varies substantially across states, with more comprehensive dental benefits associated with greater use of preventive dental care (46). A 2026 mixed-methods systematic review of 55 studies from 24 countries identified safety concerns, cost, lack of insurance, and poor referral systems as prominent barriers to dental care utilization during pregnancy (47). Current guidelines support dental treatment safety during pregnancy and encourage oral health assessment in prenatal settings (32, 42, 43). Interprofessional education and collaborative care models may strengthen integration between dental, medical, and maternal-child healthcare systems (42). Broader public health strategies, including policies addressing sugar consumption, fluoride exposure, and integration of oral health into maternal-child health initiatives, may further support population-level prevention (5, 42). Future research should use implementation science approaches to evaluate how maternal-centered strategies can be adapted across healthcare systems, populations, and resource settings (7).

### 5.3 Limitations of the framework

Limitations of this framework warrant acknowledgment. The Three-Dimensional Maternal Axis has not yet been empirically validated as an integrated model, much of the supporting evidence derives from observational studies limiting causal inference, and the relative contribution of each axis to ECC risk remains unquantified. The framework centers maternal influence, which should not be interpreted as assigning responsibility to mothers: structural determinants including poverty, healthcare access, and policy environments operate upstream of individual maternal factors and fundamentally shape the conditions within which all three axes function.

## 6 Conclusion

Accumulating evidence demonstrates that maternal biological, behavioral, and developmental factors shape ECC susceptibility during critical periods of early life, yet current preventive strategies remain largely centered on the child after tooth eruption, leaving the earliest and most formative modifiable influences unaddressed.

If ECC risk is maternally mediated across multiple dimensions, then prevention must be maternally informed across those same dimensions. Systematically mapping prevention priorities across the first 1,000 days, from conception through complementary feeding and first dentition, represents both the logical next step for this framework and a tractable foundation for future research and policy initiatives targeting the developmental origins of oral disease.

## Data Availability

The original contributions presented in the study are included in the article/Supplementary Material, further inquiries can be directed to the corresponding author.
